# Sepsis and the theory of relativity: measuring a moving target with a moving measuring stick

**DOI:** 10.1186/s13054-016-1559-z

**Published:** 2016-11-21

**Authors:** Michael Klompas, Chanu Rhee

**Affiliations:** 1 Department of Population Medicine, Harvard Medical School and Harvard Pilgrim Health Care Institute, 401 Park Street, Suite 401, Boston, MA 02215 USA; 2 Department of Medicine, Brigham and Women’s Hospital, Boston, MA USA

**Keywords:** Sepsis, Epidemiology, Administrative data, Surveillance

Sepsis is the subject of intense interest for clinicians, policy makers, patients, and researchers. Stakeholders around the world are striving to improve sepsis awareness and quality of care. The Surviving Sepsis Campaign, World Sepsis Day, and mandatory reporting rules are but a few examples of ongoing initiatives. All sepsis stakeholders need accurate data on sepsis incidence, characteristics, outcomes, and whether these are changing over time. Without these it is almost impossible to plan appropriately or to know whether our efforts to prevent sepsis and improve care are bearing fruit.

The most common source for sepsis surveillance is hospital discharge codes. Their advantages include widespread availability and low cost. At first blush they also appear relatively straightforward to analyze. On closer inspection, however, they are fraught with subtleties and uncertainties.

There are two prevailing strategies to identify sepsis using diagnosis codes: 1) identify patients with explicit codes for severe sepsis and septic shock, or 2) seek patients with concurrent discharge codes for infection and organ dysfunction, the so-called “implicit” method. The second strategy has obvious limitations. Investigators have proposed at least 5 different sets of infection and diagnosis codes to identify sepsis. Not surprisingly, different methods suggest very different sepsis rates [1]. Moreover, simultaneous infection and organ dysfunction codes at discharge do not reliably imply that organ dysfunction was due to infection.

Explicit sepsis codes also have their limitations. They were only added to the International Classification of Disease (ICD9-CM) lexicon in 2002. Despite the passage of almost 15 years since their release, clinicians and coders are still becoming familiar with them and only gradually introducing them into practice.

A recent study in *Critical Care* by Bouza et al. elucidated the uptake rate of explicit diagnosis codes using data from the Spanish national hospital discharge database for the years 2006-2011 [2]. They queried the database for sepsis using the explicit and one implicit strategy in a mutually exclusive fashion. The fraction of total cases assigned explicit codes rose from 51 % in 2006 to 64 % in 2011. Patients assigned explicit codes were sicker than patients with implicit codes alone: almost twice as many had mechanical ventilation codes and ten times as many had cardiovascular dysfunction codes. The in-hospital mortality rate for patients with explicit codes was almost double that of patients with implicit codes alone (55 % vs 29 %).

This study has important implications for stakeholders trying to understand sepsis epidemiology through code-based analyses. It is clear that one cannot consider cases identified using explicit codes interchangeably with those identified through implicit codes. It is also clear that tracking sepsis using explicit codes alone provides an incomplete and changing picture of the septic population. One might wonder, however, whether it’s feasible to track sepsis adequately using the combined population of patients with either explicit or implicit codes?

To answer this question we need to understand more about the reliability of explicit and implicit codes. Do all patients with these codes have clinically-confirmed sepsis? Do these codes collectively capture all patients with sepsis or do some cases receive neither explicit nor implicit codes? Prior work suggests that the positive predictive value is high for explicit sepsis codes but only moderate for implicit codes [3]. Implicit codes identify more patients with sepsis than explicit codes but most critically, both strategies miss large numbers of patients [3, 4]. Moreover, both explicit and implicit codes are being used more frequently over time and are thus capturing an increasing fraction of the septic population [4–8].

Bouza et al. note that increases in coding rates in Spain are probably not being driven by financial incentives because Spain has a universal health care system [2]. This still allows for the possibility, however, that clinicians and hospitals are coding more patients over time in response to the many campaigns to improve sepsis awareness and recognition and as a part of hospitals’ due diligence to improve the fidelity of coding [9].

The rise in coding rates creates a major dilemma when trying to track sepsis rates and outcomes over time. Are higher rates due to more disease, more recognition, more complete coding, or some combination of these? Are lower mortality rates due to better care, better capture of more subtle cases, or both?

More deeply, neither the explicit nor the implicit strategies overcome the ongoing challenge of sepsis classification. The new Sepsis-3 consensus definitions provide a useful conceptual definition for sepsis (“life-threatening organ dysfunction caused by a dysregulated host response to infection”) but still leave clinicians with the difficult tasks of deciding whether a given patient is infected and whether organ dysfunction is attributable to infection [10, 11]. Sometimes the answers to these questions are obvious (for example, a previously healthy patient presenting with fever, headache, and hypotension who is found to have meningococcal meningitis) but more often these questions are subtle (for example, an elderly patient with a history of congestive heart failure and dementia presenting with low grade fever, confusion, shortness of breath, and a rise in serum creatinine). Thoughtful clinicians often disagree about these more subtle cases [12]. And retrospective reviews suggest that up to 40 % of patients admitted to intensive care for sepsis treatment may not be infected after all [13].

In sum, continuing efforts to improve sepsis recognition, ongoing changes in the ways clinicians and hospitals assign codes for sepsis and organ dysfunction, and persistent uncertainties about sepsis diagnosis make sepsis surveillance using diagnosis codes incredibly challenging. Trying to track sepsis using diagnosis codes is like trying to measure a moving and uncertain target with a moving and uncertain measuring stick. We desperately need better tools for sepsis diagnosis and surveillance. One possibility is to measure sepsis using electronic clinical data rather than diagnosis codes. One can impute suspected infection from microbiology and antibiotic orders, and organ dysfunction from laboratory tests, procedures (such as mechanical ventilation), and medications (such as vasopressors) [5, 6]. This will not solve the problems of diagnostic uncertainty and changing thresholds to diagnose and treat sepsis, but it might at least mitigate the effect of changing coding patterns over time.
